# A step towards predicting what the future holds for those born extremely preterm

**DOI:** 10.1038/s41390-024-03627-0

**Published:** 2024-10-09

**Authors:** Deanne K. Thompson, Claire E. Kelly

**Affiliations:** 1 Victorian Infant Brain Studies, Murdoch Children’s Research Institute, Parkville, VIC 3052 Australia; 2 Developmental Imaging, Murdoch Children’s Research Institute, Parkville, VIC 3052 Australia; 3 Department of Paediatrics, The University of Melbourne, Parkville, VIC 3052 Australia; 4 Turner Institute for Brain and Mental Health, School of Psychological Sciences, Faculty of Medicine Nursing and Health Sciences, Monash University, Clayton, VIC 3800 Australia

## Abstract

This commentary provides a summary and discussion of the recently published study in Paediatric Research by Hedvig Kvanta et al., “Brain volumes and cortical thickness and associations with cognition in children born extremely preterm”.

Children born extremely preterm (EP: <28 weeks’ gestation) are at higher risk than their peers born at term for a range of neurodevelopmental difficulties, including motor, cognitive, and behavioural challenges.^1^ Multiple factors may influence neurodevelopmental outcomes throughout the development of those born EP. Predicting outcomes for those born EP remains complex and challenging, and could utilise information from perinatal risk factors, neuroimaging findings, early developmental assessments, and social and environmental influences.^2^

While brain volume,^3^ cortical folding measures,^4^ and diffusion tensor imaging^5^ have previously been associated with neurodevelopmental outcomes in young children born EP, a full understanding of the long-term functional significance of brain abnormalities detected using magnetic resonance imaging (MRI) is still lacking.^2^ Kvanta et al. expand this knowledge base by providing a detailed investigation of associations between MRI and neurodevelopmental outcome in a rare longitudinal long-term study of EP infants. They followed up an EP infant cohort for 12 years, whereas in the past outcome associations have less frequently been assessed beyond early childhood.

Of interest, a previous study by this group reported some of the regional brain MRI findings that differ between EP and full-term children using a neuroimaging technique called voxel-based morphometry (VBM). Volumetric decreases were found in the temporal lobes, precuneus gyri, and anterior cingulum for children born EP at age 10 years, while volumetric increases were observed in the posterior cingulum and occipital lobe.^6^ Their current study found that in 42 EP and 29 full-term control children, larger total grey and white matter brain volumes at 10 years of age were associated with higher cognition at 12 years of age, including full-scale intelligence quotient (FSIQ) and visual spatial, fluid reasoning, working memory, and processing speed index scores, but not verbal comprehension scores from the Wechsler Intelligence Scale for Children (WISC-V). These positive associations between brain volumes and cognitive outcomes were detected in both the EP and control groups, with little evidence that the associations differed between groups or sexes (i.e., no significant group or sex interactions were found). Negative associations between right mean cortical thickness at age 10 years and both FSIQ and visual spatial index at age 12 years in the whole EP and full-term cohort did not survive multiple comparison correction, and there were no group interactions for these associations. On regional VBM analyses, higher left and right insula volume at age 10 years was associated with better FSIQ scores at age 12 years in the EP children, when adjusted for sex, age at MRI and maternal education, but there were no significant VBM results for the full-term controls or for white matter volumes. There were no significant associations between EP brain volumes at term-equivalent age (*n* = 25) or longitudinal volumetric growth (10 years – term-equivalent age, *n* = 20) and cognitive scores at age 12 years. However, of note, the EP infants included in this study were a relatively healthy, lower risk group, where those with severe brain injury or abnormality were excluded.

While prior studies suggest white matter appears to be most vulnerable to injury and growth disruptions related to preterm birth,^7^ the current findings highlight that both grey and white matter abnormalities may be important for neurodevelopmental functioning following EP birth. Kvanta et. al. suggested the similar associations of grey and white matter with cognitive outcomes relates to the highly connected, inter-dependent nature of these tissues that form networks to support neurodevelopmental functioning. While brain volume was positively associated with outcomes, cortical thickness was negatively associated with outcomes. These opposing relationships may relate to known different developmental trajectories during childhood of brain volume and cortical thickness, where cortical thickness has a relatively earlier peak and decline, with cortical thinning occurring from earlier in childhood.^8,9^ Variations in brain volume and cortical thickness measurements may be driven by multiple underlying neurobiological factors (e.g. reorganization of dendritic arbour and increases in pericortical myelination), which could in turn drive differences in cognitive outcomes. Microstructural properties of the brain and their relationship to cognitive outcomes could in future be further probed using diffusion weighted imaging. Associations between global grey and white matter and cognition were similar for both EP and FT children, possibly due to individuals in both groups having a similar gap in MRI and cognitive outcome, where the EP group would generally have both reduced brain MRI measures of structural organisation and cognition.

This study took a thorough approach to examining brain structure, including both volume and thickness at a global level and at the voxel level for volume. Future analyses could include even more detailed examination of the cortical surface, via additional surface area and gyrification indices combined with parcellated regions of interest (e.g. from standard atlases such as the Desikan-Killiany (DK) or Desikan-Killiany-Tourville (DKT) atlases available within software packages such as FreeSurfer), to determine more specific brain regions and properties related to specific cognitive domains in this population. On that note, while regional associations with FSIQ were conducted using VBM for this study, it would have also been of interest to investigate the five index scores from the WISC-V in relation to the VBM technique.

While the study found that global white and grey matter volume was important for cognition, it also identified a potential specific neurological marker for poor neurodevelopment in children born EP, insula volume. It would be important for other studies to replicate this finding, particularly in *infant* EP populations, as there is potentially greater clinical utility of infant MRI for predicting later outcomes, as earlier detection means earlier intervention is possible. Kvanta et al. found that childhood MRI (age 10 years) was more strongly related to neurodevelopmental outcomes than infant MRI, however sample size limitations at the infant timepoint may have contributed to insufficient power to detect these differences. Other possible contributions to this finding include differences in the MRI protocol and MRI processing between timepoints and the use of the EP group only at the term-equivalent timepoint. It was commendable that the authors associated longitudinal MRI measures with cognitive outcomes, however the longitudinal EP sample was small (*n* = 20) and of lower risk, and therefore may have been underpowered to find meaningful associations. Further investigation would be beneficial to more fully understand associations between longitudinal brain growth and cognition in children born preterm. It remains to be seen if the larger literature on very preterm children could be extrapolated to EP children in terms of similarities in brain MRI associations with cognition. Future studies directly comparing such associations between EP, very preterm and late preterm groups would be valuable.

In summary, this study improves understanding of the relationships between MRI and cognitive outcomes in an important longitudinal, long-term, EP sample. Future work could include larger-scale datasets, more longitudinal studies, and even finer-scale surface measures, and incorporate multiple modalities such as structural, diffusion, and resting state neuroimaging to further improve understanding of the neurological basis of cognitive outcomes in preterm populations. While advanced analyses of brain MRI scans hold great promise for detecting early predictors of neurodevelopmental outcomes for children born EP, the cost, expertise and time required to conduct these analyses limit their clinical utility. A way of using clinically acquired scans to quickly and automatically assess MRI scans may be valuable. Machine learning and deep learning algorithms^10^ provide a promising way ahead for this purpose, particularly using brain MRI in combination with other perinatal predictors (such as birthweight, gestational age, bronchopulmonary dysplasia, infection, and brain injury) and social and environmental factors (such as parental education and socio-economic status). This may give the best chance of identifying an individual child’s risk for abnormal long-term outcomes to target the most appropriate intervention strategy based upon that risk.^2^ This could have the potential to move towards personalised medicine, with the aim of improving the lives of high-risk infants such as those born EP.
